# Risk of secondary appendiceal tumours after colorectal cancer surgery: nationwide Swiss registry study

**DOI:** 10.1093/bjsopen/zraf147

**Published:** 2026-01-13

**Authors:** Jeremy Meyer, Elin Meyer, Evelyne Fournier, Emilie Liot, Guillaume Meurette, Thibaud Koessler, Christian Toso, Justin Davies, James Wheeler, Katharina Staehelin, Elisabetta Rapiti, Frédéric Ris, Lea Wildisen

**Affiliations:** Division of Digestive Surgery, Department of Surgery, University Hospitals of Geneva, Geneva, Switzerland; Medical School, University of Geneva, Geneva, Switzerland; Division of Digestive Surgery, Department of Surgery, University Hospitals of Geneva, Geneva, Switzerland; Medical School, University of Geneva, Geneva, Switzerland; Medical School, University of Geneva, Geneva, Switzerland; Registre Genevois des Tumeurs, University of Geneva, Geneva, Switzerland; Division of Digestive Surgery, Department of Surgery, University Hospitals of Geneva, Geneva, Switzerland; Medical School, University of Geneva, Geneva, Switzerland; Division of Digestive Surgery, Department of Surgery, University Hospitals of Geneva, Geneva, Switzerland; Medical School, University of Geneva, Geneva, Switzerland; Medical School, University of Geneva, Geneva, Switzerland; Department of Oncology, University Hospitals of Geneva, Geneva, Switzerland; Division of Digestive Surgery, Department of Surgery, University Hospitals of Geneva, Geneva, Switzerland; Medical School, University of Geneva, Geneva, Switzerland; Addenbrooke’s Hospital, Cambridge University Hospitals NHS Foundation Trust, Cambridge, UK; Colorectal Surgery Unit, University of Cambridge, Cambridge, UK; Addenbrooke’s Hospital, Cambridge University Hospitals NHS Foundation Trust, Cambridge, UK; National Agency for Cancer Registration (NACR), Zürich, Switzerland; Medical School, University of Geneva, Geneva, Switzerland; Registre Genevois des Tumeurs, University of Geneva, Geneva, Switzerland; Division of Digestive Surgery, Department of Surgery, University Hospitals of Geneva, Geneva, Switzerland; Medical School, University of Geneva, Geneva, Switzerland; National Agency for Cancer Registration (NACR), Zürich, Switzerland

**Keywords:** appendix cancer, epidemiology, adenocarcinoma, NET

The appendix can harbour synchronous or metachronous tumours in patients with colorectal cancer (CRC)^1^, yet prophylactic appendicectomy is not routinely recommended. This study assessed the incidence of secondary appendiceal tumours after CRC surgery using Swiss population-based data.

The Swiss national cancer data set (National Agency for Cancer Registration) was analysed for the period 2009–2021^2^. Adults with a first primary colorectal adenocarcinoma who underwent surgical resection in Switzerland were included. Patients with previous CRC, primary appendiceal cancer, or age < 18 years were excluded. Standardized incidence ratios (SIRs) compared the risk of second primary appendiceal tumours with that of the general Swiss population, adjusting for age, sex, and calendar year. Tumours diagnosed within 4 months of the CRC diagnosis were deemed synchronous; later diagnoses were considered metachronous^3^.

Among 25 714 patients who underwent CRC surgery (mean age 69 years, 44% female), 56 developed a second primary appendiceal tumour during 133 227 person-years of follow-up (incidence 42 per 100 000 person-years; *Tables S1–S3*). Most were neuroendocrine tumours (93%) and were detected within 4 months of CRC diagnosis (86%). Compared with the background Swiss population (incidence 1.83 per 100 000 person-years), the risk in this cohort was markedly increased (SIR 19.8; 95% confidence interval (c.i.) 15.0 to 25.4).

Sensitivity analyses confirmed elevated risk when limited to patients with metachronous tumours (8 patients; SIR 2.8, 95% c.i. 1.3 to 5.3) and when excluding patients who underwent right hemicolectomy (SIR 12.4; 95% c.i. 8 to 18.2). Excluding neuroendocrine tumours still yielded a slightly raised risk (SIR 3.2; 95% c.i. 1.0 to 7.4; *Table 1*).

**Table 1:** Estimation of the risk for appendiceal tumours in patients operated on for CRC in Switzerland

	Observed no. of appendiceal tumours	Person-years at risk	Expected no. of appendiceal tumours	Age-standardized incidence ratio*	*P*
Reference population	1707	93 152 298		1	
**All histologies**					
Synchronous and metachronous					
Patients operated on for CRC	56	133 227	2.83	19.76 (15.03, 25.40)	< 0.001
Patients operated on for left-sided CRC	23	85 827	1.86	12.38 (8.00, 18.15)	< 0.001
**Metachronous appendiceal tumours**					
Metachronous					
Patients operated on for CRC	8	133 466	2.84	2.82 (1.29, 5.25)	0.003
Patients operated on for left-sided CRC	7	85 922	1.86	3.76 (1.62, 7.28)	< 0.001
**Non-NET tumours**					
Synchronous and metachronous					
Patients operated on for CRC	4	131 010	1.26	3.17 (0.98, 7.37)	0.021

^*^Values in parentheses are 95% confidence intervals. CRC, colorectal cancer. NET, neuroendocrine tumours.

Overall, this nationwide analysis showed that patients undergoing CRC surgery have a nearly 20-fold higher incidence of appendiceal tumours than the Swiss population, and higher than described in other populations^4,5^. Most were early-stage neuroendocrine tumours of uncertain clinical significance. Although synchronous appendicectomy adds minimal operative risk in elective colorectal resections, the absolute benefit would be considered small, with prophylactic removal preventing appendiceal tumours in only 0.2% of the present cohort.

Limitations of this study include the lack of individual appendicectomy data and possible detection bias from intensified postoperative surveillance.

## Supplementary Material

zraf147_Supplementary_Data

## Data Availability

Data are available upon reasonable request to the corresponding author.
