# Supplementary material for: Risk of secondary appendiceal tumours after colorectal cancer surgery: nationwide Swiss registry study
Source: BJS Open. 2026 Jan 13;10(1):zraf147. doi: 10.1093/bjsopen/zraf147 (PMC12798808; doi:10.1093/bjsopen/zraf147)
Supplement: zraf147_Supplementary_Data [file zraf147_supplementary_data.docx]

**Risk of Secondary Appendiceal Tumors After Colorectal Cancer Surgery: Evidence From a Nationwide Swiss Registry Study**

Jeremy Meyer, MD, PhD ^1,2^, Elin Meyer, MD, PhD ^1,2^, Evelyne Fournier ^2,3^, Emilie Liot, MD ^1,2^, Guillaume Meurette, MD, PhD ^1,2^, Thibaud Koessler, MD, PhD ^2,4^, Christian Toso, MD, PhD ^1,2^, Justin Davies, MA, MChir ^5,6^, James Wheeler, MD ^5^, Katharina Staehelin, MD, MPH ^7^, Elisabetta Rapiti, MD, PhD 2,^3^, Frédéric Ris, MD ^1,2^, Lea Wildisen, PhD ^7^

1. Division of Digestive Surgery

Department of Surgery

University Hospitals of Geneva

Rue Gabrielle-Perret-Gentil 4

1211 Genève 14

Switzerland

2. Medical School

University of Geneva

Rue Michel-Servet 1

1206 Genève

Switzerland

3. Registre Genevois des Tumeurs

University of Geneva

Rue Michel-Servet 1

1206 Genève

Switzerland

4. Department of Oncology
University Hospitals of Geneva

Rue Gabrielle-Perret-Gentil 4

1211 Genève 14

Switzerland

5. Addenbrooke’s Hospital

Cambridge University Hospitals NHS Foundation Trust

CB2 0QQ Cambridge

United Kingdom

6. University of Cambridge
Cambridge
United Kingdom

7. National Agency for Cancer Registration (NACR)

Zürich

Switzerland

**Corresponding author:**

Jeremy Meyer, MD, MD-PhD

Division of Digestive Surgery

University Hospitals of Geneva

Rue Gabrielle-Perret-Gentil 4

1211 Genève 14

Switzerland

E-mail: jeremy.meyer@hug.ch

Phone: +41.22.379.52.52

**Supplementary Figures and Tables**

**Table 1: Characteristics of all colorectal cancer diagnoses in Switzerland between January 2009 and December 2021**

|  | **Colorectal cancer (adenocarcinoma)** |
| --- | --- |
| Total, n (%) | 38493 (100) |
| Female, n (%) | 16577 (43.1) |
| Localization (ICD-10 code), n (%) |  |
| *Cecum (C180)* | 4735 (12.3) |
| *Appendix (C181)* | 431 (1.1)* |
| *Ascending colon (C182)* | 4830 (12.5) |
| *Hepatic flexure (C183)* | 1417 (3.7) |
| *Transverse colon (C184)* | 2052 (5.3) |
| *Splenic flexure (C185)* | 870 (2.3) |
| *Descending colon (C186)* | 1595 (4.1) |
| *Sigmoid colon (C187)* | 9092 (23.6) |
| *Overlapping lesion of colon (C188)* | 286 (0.7) |
| *Colon, unspecified (C189)* | 409 (1.1) |
| *Rectosigmoid junction (C19)* | 2313 (6) |
| *Rectum (C20)* | 10463 (27.2) |
| TNM Stage UICC, n(%)  Stage I  Stage II  Stage III  Stage IV  Missing | 6266 (16.3)  11363 (29.5)  9552 (24.8)  8430 (21.9)  2882 (7.5) |

* Excluded from further analyses

|  | Patients who underwent surgery for colorectal cancer (at-risk population) | Patients who underwent surgery for left-sided colorectal cancer* |
| --- | --- | --- |
| Total, n (%) | 25714 (100) | 15786 (100) |
| Female, n (%) | 11248 (43.7) | 6259 (39.6) |
| Median age, years (IQR) | 70.7 (61.2-78.9) | 68.7 (59.5-77.7) |
| Type (ICD-10 code), n (%) |  |  |
| *Cecum (C180)* | *3199 (12.4)* | *-* |
| *Ascending colon (C182)* | *3352 (13)* | *-* |
| *Hepatic flexure (C183)* | *942 (3.7)* | *-* |
| *Transverse colon (C184)* | *1380 (5.4)* | *-* |
| *Splenic flexure (C185)* | *593 (2.3)* | *-* |
| *Descending colon (C186)* | *1123 (4.4)* | *1117 (7.1)* |
| *Sigmoid colon (C187)*  *Overlapping lesion of colon (C188)* | *6454 (25.1)*  *212 (0.8)* | *6423 (40.7)* |
| *Colon, unspecified (C189)* | *168 (0.7)* | *-* |
| *Rectosigmoid junction (C19)* | *1539 (6)* | *1525 (9.7)* |
| *Rectum (C20)* | *6752 (26.3)* | *6722 (42.6)* |
| TNM Stage UICC, n (%)  *Stage I*  *Stage II*  *Stage III*  *Stage IV*  Missing | 4844 (18.8)  8760 (34.1)  7139 (27.8)  4298 (16.7)  673 (2.6) | 3225 (20.4)  5123 (32.5)  4339 (27.5)  2551 (16.2)  548 (3.5) |

**Table 2: Characteristics of patients who underwent surgery for colorectal cancer in Switzerland between January 2009 and December 2021**

*Excluding right hemicolectomy, total and subtotal colectomy

**Table 3: Incidental cases of appendix tumors in patients who underwent surgery for colorectal cancer and in the general population in Switzerland between January 2009 and December 2021**

|  | **Primary Appendix Tumors**  **(in the reference population)** | | **Second Primary Appendix Tumors**  **(in the at-risk population)*** | |
| --- | --- | --- | --- | --- |
| **Demographics** |  |  |  |  |
| Total, n | 1704 |  | 56 |  |
| Female, % | 54.7 |  | 46.4 |  |
| Median age, years (IQR) | 55.4 (38.2-68.4) |  | 67.4 (57.0–77.0) |  |
| **ICD 10 group** | **N** | **%** | **N** | **%** |
| Appendix (C181) | 1704 | 100 | 56 | 100 |
| **Synchronicity** | **N** | **%** | **N** | **%** |
| Synchronous | - | - | 48 | 85,71 |
| Metachronous | - | - | 8 | 14,29 |
| **ICD-O-3 Morphology** | **N** | **%** | **N** | **%** |
| 8000 | 5 | 0.29 | 0 | 0 |
| 8010 | 3 | 0.18 | 0 | 0 |
| 8013 | 1 | 0.06 | 0 | 0 |
| 8140 | 174 | 10.21 | 2 | 3.57 |
| 8144 | 5 | 0.29 | 0 | 0 |
| 8210 | 5 | 0.29 | 0 | 0 |
| 8211 | 1 | 0.06 | 0 | 0 |
| 8240** | 865 | 50.76 | 46 | 82.14 |
| 8241** | 1 | 0.06 | 0 | 0 |
| 8243** | 159 | 9.33 | 2 | 3.57 |
| 8244** | 100 | 5.87 | 1 | 1.79 |
| 8245** | 13 | 0.76 | 0 | 0 |
| 8246** | 45 | 2.64 | 2 | 3.57 |
| 8249** | 35 | 2.05 | 1 | 1.79 |
| 8261 | 1 | 0.06 | 0 | 0 |
| 8263 | 8 | 0.47 | 0 | 0 |
| 8441 | 1 | 0.06 | 0 | 0 |
| 8480 | 204 | 11.97 | 0 | 0 |
| 8481 | 17 | 1.00 | 0 | 0 |
| 8490 | 47 | 2.76 | 0 | 0 |
| 8574 | 4 | 0.23 | 0 | 0 |
| 8936 | 3 | 0.18 | 2 | 3.57 |
| missing | 7 | 0.41 | 0 | 0 |
| **Stage** | **N** | **%** | **N** | **%** |
| I | 85 | 5 | 4 | 7.1 |
| II | 275 | 16.1 | 1 | 1.8 |
| III | 87 | 5.1 | 1 | 1.8 |
| IV | 225 | 13.2 | 1 | 1.8 |
| missing | 1032 | 60.6 | 50 | 89.3 |
| **Localisation of primary colorectal cancer** | **N** | **%** | **N** | **%** |
| Cecum (C180) | - | - | 12 | 21.43 |
| Ascending colon (C182) | - | - | 13 | 23.21 |
| Hepatic flexure (C183) | - | - | 2 | 3.57 |
| Transverse colon (C184) | - | - | 4 | 7.14 |
| Splenic flexure (C185) | - | - | 1 | 1.79 |
| Descending colon (C186) | - | - | 1 | 1.79 |
| Sigmoid colon (C187) | - | - | 7 | 12.50 |
| Colon, unspecified (C189) | - | - | 1 | 1.79 |
| Rectosigmoid junction (C19) | - | - | 4 | 7.14 |
| Rectum (C20) | - | - | 11 | 19.64 |

*Patients who were operated for colorectal cancer

** ICD-O-3.2 codes which were considered as neuroendocrine tumors.
